# NK3.3-Derived Extracellular Vesicles Penetrate and Selectively Kill Treatment-Resistant Tumor Cells

**DOI:** 10.3390/cancers16010090

**Published:** 2023-12-23

**Authors:** Allyson McCune, Jacki Kornbluth

**Affiliations:** 1 Department of Pathology, Saint Louis University School of Medicine, St. Louis, MO 63104, USA; amccunephd@outlook.com; 2 St. Louis VA Medical Center, St. Louis, MO 63106, USA

**Keywords:** natural killer, extracellular vesicles, leukemia, breast cancer, cancer stem cells

## Abstract

**Simple Summary:**

Many cancer treatments become ineffective as tumor cells develop resistance and escape, resulting in patient relapse and tumor spread. Therefore, there is an urgent need to develop alternative approaches to target cancer and improve patient outcomes. We reported that extracellular vesicles (EVs) derived from the human natural killer (NK) cell line NK3.3 have potent tumor-killing activity, without harming normal cells. This study was designed to evaluate the ability of NK3.3EVs to overcome some of the obstacles to treatment efficacy. We used a three-dimensional breast cancer model to demonstrate that NK3.3EVs effectively penetrate and kill solid tumor cells. We developed a drug-resistant leukemia cell line and found that NK3.3EVs are highly cytotoxic to these cells. These EVs also eliminate the subpopulation of cancer cells that evade treatment and are responsible for tumor recurrence. These studies indicate that NK3.3EVs are a potential new immunotherapeutic agent for difficult-to-treat cancers.

**Abstract:**

Cancer treatments often become ineffective due to the development of tumor resistance, leading to metastasis and relapse. Treatments may also fail because of their inability to access cells deep within the tumor tissue. When this occurs, new therapeutic agents are needed. We previously reported that NK3.3EVs, extracellular vesicles (EVs) derived from the normal human natural killer (NK) cell line, NK3.3, have strong cytotoxic activity against leukemia and breast cancer cell lines, without harming normal cells. Here, we used a three-dimensional (3D) MCF7 breast cancer mammosphere model to reproduce a more physiological environment that NK3.3EVs would encounter in vivo. NK3.3EVs penetrated MCF7 mammospheres, inducing death by apoptosis. We generated an imatinib-resistant K562 chronic myeloid leukemia (CML) cell line to investigate whether NK3.3EVs were able to kill tumor cells resistant to front-line chemotherapy. NK3.3EVs were even more cytotoxic to imatinib-resistant cells than parental cells, inducing apoptosis via caspase-3/-7 activation. The small population of cancer stem cells (CSCs) within tumors also contributes to therapeutic resistance. NK3.3EVs reduced the CSC-like CD34+/CD38− subpopulation in imatinib-resistant and parental K562 cultures and decreased CSC-associated expression of tumor-promoting genes. Our results provide strong evidence that NK3.3EVs may be a potential new immunotherapeutic agent for difficult-to-treat cancers.

## 1. Introduction

A 2021 review of global data reported that there were over 23 million new cancer cases and 10 million cancer deaths, an increase of 26.3% and 20.9% since 2010, respectively, and the second leading cause of death. There are several factors that contribute to treatment failures, including the development of drug resistance and the persistence of cancer stem cells (CSCs), leading to relapse and metastasis [1]. As the incidence of cancer continues to increase, new treatments are urgently needed on a global scale [2]. Investigations of new therapeutic agents are initially conducted in vitro, in a two-dimensional culture format where tumor cells grow laterally in a monolayer across a single plane. New techniques using tumorspheres, three-dimensional (3D) clusters of solid tumor cells grown in wells that prevent surface adherence, simulate a more physiological environment. The data derived from 3D models are more representative of how treatments may affect tumor cells in vivo [3,4,5,6].

A new approach to cancer therapy under investigation is the use of extracellular vehicles (EVs)—biological nanoparticles produced by cells for the communication and distribution of cellular materials between cells. EVs are secreted by cells into the extracellular space. They are heterogeneous in size and their contents reflect their biogenesis from the cell. EVs have a structure that consists of a lipid bilayer membrane and an internal luminal space [7]. Natural killer (NK) cell-derived EVs from mice, human peripheral blood, human non-Hodgkin’s lymphoma NK cell line (NK-92), and our normal human NK cell line, NK3.3, have been evaluated for their molecular content and function. We demonstrated that NK3.3-derived EVs express typical exosomal proteins (TSG101, ALIX, CD63, and HSP70). They also contain the same cytolytic effector proteins used by NK cells to kill tumor cells, and they induce the death of tumor cells when applied to them [8,9,10,11,12,13,14,15,16,17,18,19,20,21,22]. Studies by us and others have shown that NK EVs exhibit a dose- and time-dependent cytotoxic effect in vitro against multiple cancer cell lines, including multiple myeloma, leukemia, lymphoma, breast cancer, glioblastoma, colon, ovarian, liver, melanoma, prostate, stomach, cervical, and lung [8,14,15,16,17,18,19,22]. Importantly, NK EVs do not kill normal, healthy cells, making them a potentially new cancer treatment that, unlike chemotherapy and radiotherapy, would allow patients to maintain a high quality of life.

Imatinib, a tyrosine kinase inhibitor, is used as a first-line chemotherapeutic agent for patients with chronic myeloid leukemia (CML) [23]. Imatinib inhibits the Bcr-Abl fusion protein, produced by a chromosomal translocation in CML cells. Bcr-Abl tyrosine kinase activity activates proteins associated with proliferation and metabolic activity; imatinib inhibits this activity. Mutations in the Bcr-Abl protein and other mechanisms arise over time in CML cells to render imatinib ineffective [23,24]. Another potentiator of resistance is the self-renewing, pluripotent CSC subpopulation within tumors. These cells evade treatment due in part to mutations in normal stem cell DNA and the phenotypic plasticity of more differentiated tumor cells [1,25]. Therefore, finding new treatments capable of reducing the CSC population and drug-resistant tumor cells that contribute to relapse and metastasis would be a major advance in cancer therapy.

In previous studies, we demonstrated that NK3.3EV treatment induced the death of leukemia and breast cancer cells in vitro and of triple-negative breast cancer (TNBC) cells in vivo [14]. Here, we investigated the degree to which NK3.3EVs penetrate and kill solid breast tumors in an in vitro 3D mammosphere tumor model. We also evaluated whether NK3.3EVs could circumvent resistance mechanisms to induce the death of imatinib-resistant K562 cells, a representative drug-resistant CML cell line, and eliminate the CSC-like tumor subpopulation in CML responsible for disease recurrence.

## 2. Materials and Methods

### 2.1. Cell Lines

The human NK cell line, NK3.3, was maintained in RPMI-1640 media supplemented with 10% fetal bovine serum (FBS), 1% glutamine, 1% penicillin-streptomycin, and 200 U/mL recombinant interleukin (IL)-2 (IL-2) (R&D Systems, Minneapolis, MN, USA). Media with EV-depleted FBS were used to grow NK3.3 cells for NK3.3EV isolation. To obtain EV-depleted FBS, FBS was ultracentrifuged at 118,000× *g* (Type 45 Ti rotor, Beckman Coulter, Indianapolis, IN, USA) for 20 h, then passed through a 0.22 μm filter. K562 leukemia cells (American Type Culture Collection (ATCC), Manassas, VA, USA) were cultured in RPMI-1640 media supplemented with 7.5% FBS and 1% L-glutamine. Adherent breast cancer cell lines MCF7 and MDA-MB-231 and HEK293 epithelial cells were obtained from the ATCC and human foreskin fibroblasts (HFFs) were provided by Dr. Ratna Ray, Saint Louis University. They were cultured in DMEM supplemented with 10% FBS, 1% glutamine, and 1% penicillin-streptomycin. Green fluorescent protein (GFP)-expressing MCF7 cells were generated by lipofectamine-mediated transfection of the pcDNA-GFP plasmid. After selection in G418, neomycin-resistant GFP+ cells were plated at 1 cell per well in 96-well plates to generate stable GFP+ MCF7 single-cell clones. A clone with high GFP expression was expanded and used in these experiments. All cells were incubated at 37 °C in a humidified atmosphere with 5% CO_2_. Cell lines were regularly tested for mycoplasma using the InvivoGen Mycoplasma Detection Kit (InvivoGen, San Diego, CA, USA).

### 2.2. Drug-Resistant K562 Cell Line Development

K562 cells were exposed to increasing concentrations of imatinib (TCI America, VWR, Portland, OR, USA) in RPMI-1640 media supplemented with 7.5% FBS and 1% L-glutamine, beginning with 100 nM to 500 nM in increments of 75 nM. Ten million cells were cultured in each imatinib concentration for 1–2 weeks. Depending upon the imatinib concentration, cells were cultured for up to 3 weeks before growing cells emerged. If significant cell debris accumulated, live cells were separated from debris by Histopaque-1077 density gradient centrifugation. Once cells began to grow, the imatinib concentration increased. Cells were then continuously cultured in 500 nM imatinib.

### 2.3. Sphere Formation

For mammosphere formation, adherent MCF7 breast cancer cells were washed with PBS and detached from T25 flasks with 0.25% trypsin-EDTA. Cells were resuspended in mammosphere growth media (PromoCell 3D Tumorsphere Media XF) (MilliporeSigma, Burlington, MA, USA) and immediately centrifuged at 1200 rpm for 5 min. Cell pellets were resuspended in mammosphere growth media. Single-cell suspensions were obtained by passing cells through a 100 µm, followed by a 40 µm cell strainer (BD Falcon, Fisher Scientific, Pittsburgh, PA, USA). Cells were plated at 10,000 cells/mL in ultra-low-attachment (ULA) 12-well flat-bottom plates (Corning, Corning, NY, USA) and incubated at 37 °C and 5% CO_2_ for up to 7 days to allow mammosphere formation prior to EV treatment. Half the volume of media was replaced with fresh media every 3–4 days.

For 3D sphere cultures, cells were detached from flasks, and single-cell suspensions were made as described above. HEK293 cells were plated at 250 cells/well, and MCF7 cells were plated at 500 cells/well in 200 μL DMEM with 10% FBS in ULA 96-well U-bottom plates (Corning). Plates were centrifuged at 1000 rpm for 10 min, then incubated at 37 °C and 5% CO_2_. Spheres formed after 3 days. On day 3, half the volume of media was replaced with media containing the various treatments.

### 2.4. EV Generation and Isolation

EV isolation and characterization were previously described [14,15]. Briefly, NK3.3 cells were cultured overnight in RPMI-1640 media with 3% EV-depleted FBS and 200 U IL-2/mL and then treated with phorbol myristate acetate and ionomycin for 5 h to augment EV release. HEK293 cells were grown to 90% confluency over 48 h in DMEM and 3% EV-depleted FBS. NK3.3 cell suspensions and HEK293 culture media were centrifuged at 300× *g* for 10 min to pellet cells and debris. Supernatants were removed, centrifuged again at 2000× *g* for 10 min to remove smaller debris, and passed through a 0.22 μm filter. A commercial polyethylene glycol polymer for EV precipitation was added to the supernatants for a minimum of 12 h at 4 °C (ExoQuick-TC, System Biosciences, Palo Alto, CA, USA). After incubation, supernatants were centrifuged at 3000× *g* at 4 °C for 10 min per a modified ExoQuick-TC protocol. EV pellets were resuspended in phosphate-buffered saline (PBS). Protein concentrations were assessed using the BCA protein assay kit (Thermo Fisher Scientific, Waltham, MA, USA); particle numbers and sizes were determined by nanoparticle tracking analysis (NTA) (NanoSight NS3000, Malvern Panalytical, Malvern, UK). There was minimal batch-to-batch variation in EV preparations made by PEG precipitation. For both HEK293 and NK3.3 EVs, 1 μg of protein consisted of 1–2 × 10^11^ EV particles.

### 2.5. Cell Growth/Viability Assays

Cells were placed in fresh culture media 24 h before treatment. For cell viability assessed by trypan blue dye exclusion, cells were resuspended in fresh culture media at final concentrations of 5 × 10^5^ cells/mL/well for K562 cells and 1 × 10^5^ cells/mL/well for MCF7 monolayers and mammospheres. Cells were incubated with either PBS, 100 μg/mL HEK293EVs or NK3.3EVs, 500 nM imatinib, or 100 nM paclitaxel (pax) (Invitrogen, Waltham, MA, USA). Treatments prepared in PBS were added to constitute 10% of the total well volume. Cells were evaluated morphologically, and live cells were counted (after trypsin dissociation of MCF7 cells) using an automated cell counter (Countess II FL).

Cell viability and growth of adherent cell lines HEK293 and HFF were assessed by counting live cells by trypan blue dye exclusion as well as with a crystal violet staining assay. Cells seeded in 96-well flat-bottom plates at 3000/well were treated with PBS, 100 μg/mL HEK293EVs, or NK3.3EVs. At each time point, media were removed from the wells; wells were washed with PBS, and then incubated with 35 μL of 0.1% crystal violet stain (MilliporeSigma) at room temperature for 10 min. After the removal of the stain, 50 μL of lysing solution was added (0.1 M sodium citrate in 50% ethanol, pH 4.2) and incubated for 15 min. Plates were read on a BioTek Gen5 multimode microplate reader using a 590 nm filter to measure colorimetric absorbance (Agilent, Santa Clara, CA, USA).

### 2.6. Flow Cytometry

Apoptosis/cell death was determined by staining cells with fluorescent PE-Annexin V (#640908, BioLegend, San Diego, CA, USA) and 7-AAD (#559925, BD Biosciences, San Jose, CA, USA) or V450 ghost dye (#13-0863-T100, TONBO Biosciences, San Diego, CA, USA). CSC phenotyping was performed by incubating K562 cells in staining buffer (PBS and 1% bovine serum albumin) with fluorescent antibodies AF488 anti-CD34 (#343517) and APC anti-CD38 (#303509) (Biolegend). All assays were performed following manufacturers’ protocols. Cells were analyzed using an LSRII flow cytometer (BD Biosciences). Data were analyzed with FlowJo software 10.7.2.

### 2.7. Caspase-3/7 Activity Assay 

Parent and imatinib-resistant K562 cells were treated with 100 μg/mL HEK293EVs or NK3.3EVs, 500 nM imatinib, or the apoptosis inducer staurosporine at 2.5 μM (Millipore Sigma), in 96-well plates seeded with 50,000 cells/well in 100 μL of total volume. Treatments were performed in triplicate for each condition and time point. Caspase-3/7 activity was quantitated using the Caspase-Glo 3/7 assay system (Promega, Madison, WI. USA) following the kit protocol adapted for 5000 cells in 30 μL volume per reaction in white 384-well plates. Luminescence was measured using the BioTek Gen5 multimode microplate reader.

### 2.8. RNA Analysis

For qPCR analysis of CSC-associated genes, 3 × 10^5^ parent and imatinib-resistant K562 cells were treated with PBS or 100 μg/mL NK3.3EVs for 24 h. RNA was prepared with the RNeasy Mini Kit (Qiagen, Germantown, MD, USA) following the manufacturer’s protocol. RNA concentrations were determined using a NanoDrop spectrophotometer (Thermo Fisher Scientific). cDNA was synthesized from 100 ng RNA using the Invitrogen cDNA kit. Samples were placed in a thermocycler with a programmed cycle of 42 °C for 15 min, followed by 95 °C incubation for 5 min, and then diluted with RNAse-free water. Forward (F) and reverse (R) primers for hc-Myc F: 5′ CCTGGTGCTCCATGAGGAGAC, R: 5′ CAGACTCTGACCTTTTGCCAGG (IDT); pan-hLDHa F: 5′ GGTTTGTAAAATCCACAGCTATATCC, R: 5′ GTGACTCACTGGGAAAAAATGTTGG (IDT); SOX2 F: TACAGCATGTCCTACTCGCAG, R: GAGGAAGAGGTAACCACAGGG; 18S rRNA housekeeping control F: 5′ CCATCGAACGTCTGCCCTA, R: 5′ TCACCCGTGGTCACCATG (MilliporeSigma); OCT4 (gifted by Dr. Ratna Ray) were added to 2 μL aliquots of cDNA samples and SYBR Green Master Mix (Invitrogen) to make 10 μL total sample volume per replicate. Samples were run using the Applied Biosystems 7500 real-time PCR system. 7500 Software v 2.3-derived CT values and 2(^−^ΔΔCT) were calculated to determine fold changes between untreated and NK3.3EV-treated parent and resistant K562 cells. Three replicates were run in each of three experiments.

### 2.9. EV Uptake Assays

To detect EV uptake, EVs were first labeled with the lipophilic red fluorescent dioctadecyl (DiD) Vybrant stain (Invitrogen). A total of 100 μg of HEK293EVs and NK3.3EVs in 50 μL PBS were each labeled with 1 μL of 50 μM DiD stain for 20 min at 37 °C. After incubation, 450 μL PBS was added to the EVs, followed by 167 μL ExoQuick. Suspensions were mixed gently and incubated for 2 h at 4 °C. Tubes were centrifuged at 10,000 rpm for 10 min, supernatants aspirated, and EV pellets resuspended in 50 μL PBS and applied to cells. Monolayer and mammosphere cells were seeded as described above. After EV treatment, cells and spheres were gently washed to remove non-internalized EVs. Control cells were incubated with DiD alone. HFF and MDA-MB-231 monolayer cells and MCF7 mammospheres were imaged in situ with a Leica DMI4000B inverted fluorescence microscope. MCF7 U-bottom spheres were imaged in situ with the Leica inverted fluorescence microscope, a Bio-Rad ZOE fluorescent cell imager, and inverted phase microscope.

EV-treated HEK293 and GFP+ MCF7 spheres were removed from wells, washed with PBS, fixed in 100 μL 4% paraformaldehyde (PFA) for 30 min, and then transferred in PFA to covered glass chamber slides. Images were obtained by Leica spinning-disk or confocal fluorescence microscopes in the Research Microscopy and Histology Core, Saint Louis University.

### 2.10. Statistical Analyses

All data were recorded and analyzed in Microsoft Excel (Office 365) and expressed as mean ± standard error (SE). Statistical significance was determined at *p* ≤ 0.05 using Student’s *t*-test with unequal variance.

## 3. Results

### 3.1. NK3.3-Derived EVs Kill Breast Cancer Cells in Monolayer and Mammosphere Cultures

**Figure 1:**
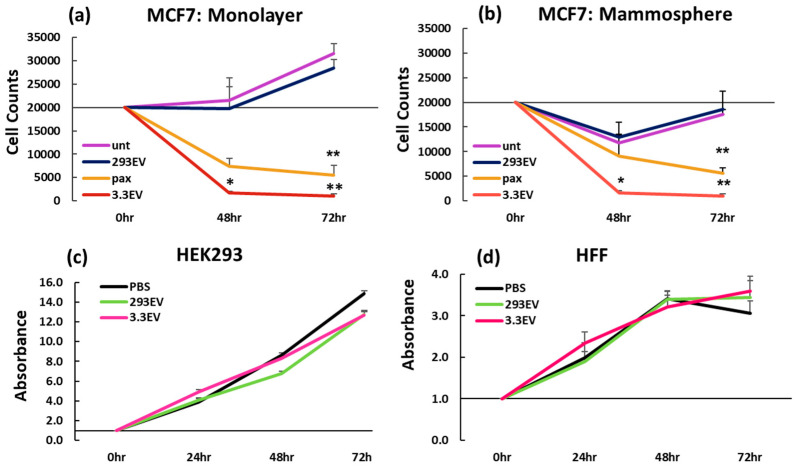
NK3.3EVs kill MCF7 cells in monolayer and mammosphere cultures but do not affect HEK293 or normal fibroblast growth or viability. After treatment with PBS (unt), 100 μg/mL HEK293EVs (293EV), 100 μg/mL NK3.3EVs (3.3EV), or 100 nM paclitaxel (pax), cell counts were performed by trypan blue dye exclusion with an automated cell counter at 48 and 72 h for MCF7 cells in (**a**) monolayers and (**b**) mammospheres. For both HEK293 and NK3.3EVs, a concentration of 100 μg/mL corresponds to ~1–2 × 10^13^ particles/mL. Mean live cell counts ± SE; n = 3. *p*-values determined by comparison of NK3.3EV-treated to HEK293EV-treated cells. * *p* ≤ 0.05, ** *p* ≤ 0.005. (**c**) HEK293 and (**d**) HFF cells were treated with PBS, 100 μg/mL HEK293EVs (293 EV), or 100 μg/mL NK3.3EVs (3.3 EV). Growth and viability were evaluated using crystal violet staining at 0, 24, 48, and 72 h. Absorbance readings at 0 h were set to 1; subsequent readings for each treatment were normalized to untreated cells. Mean absorbance ± SE; n = 3.

We previously characterized EVs isolated from NK3.3 cells [14,15]. The majority are ~135 nm in size (Figure S1) and express exosomal markers (TSG101, ALIX, CD63, and CD81). NK EVs contain the cytolytic proteins found in NK granules (granzymes A, B, perforin, and NKLAM/RNF19b), indicating that both NK granules and EVs share a common biogenesis from multivesicular bodies. We compared the ability of NK3.3EVs to kill MCF7 breast cancer cells grown as monolayers and mammospheres. MCF7 cells express estrogen and progesterone receptors and are less aggressive and invasive than TNBC [26]. Paclitaxel (pax), a first-line chemotherapeutic drug for treating breast cancer, was used as a positive control. Pax binds microtubules during mitosis, preventing their depolymerization and retraction during anaphase, leading to mitotic arrest and apoptosis [27]. EVs derived from HEK293 cells, processed in parallel with NK3.3EVs, served as a negative EV control in experiments. MCF7 mammospheres were generated in low-attachment, flat 12-well plates, where multiple spheres formed in each well. Monolayer cultures were generated in tissue culture-treated, flat 12-well plates. MCF7 cells were treated with NK3.3EVs, HEK293EVs, or pax for 48 and 72 h. Live cell counts were performed at each time point after trypsinization to remove cells from the plate surface or to dissociate spheres. Both NK3.3EV-treated monolayer cells (Figure 1a) and mammosphere cells (Figure 1b) were almost completely eliminated by 72 h. Pax treatment was less effective than NK3.3EV treatment in killing MCF7 cells in both monolayer and mammosphere cultures. HEK293EVs had no effect on the cells; HEK293EV-treated MCF7 in both monolayer and mammosphere cultures had the same growth pattern as untreated cells. 

### 3.2. NK3.3-Derived EVs Are Not Toxic to Non-Tumorigenic Cells

Monolayer cultures of HEK293 and human foreskin fibroblasts (HFFs) were treated with HEK293EVs or NK3.3EVs over 72 h. NK3.3EV treatment had no effect on the growth or viability of HEK293 cells (Figure 1c) or HFF (Figure 1d).

### 3.3. NK3.3-Derived EVs Induce Apoptosis of MCF7 Cells in Mammospheres 

**Figure 2:**
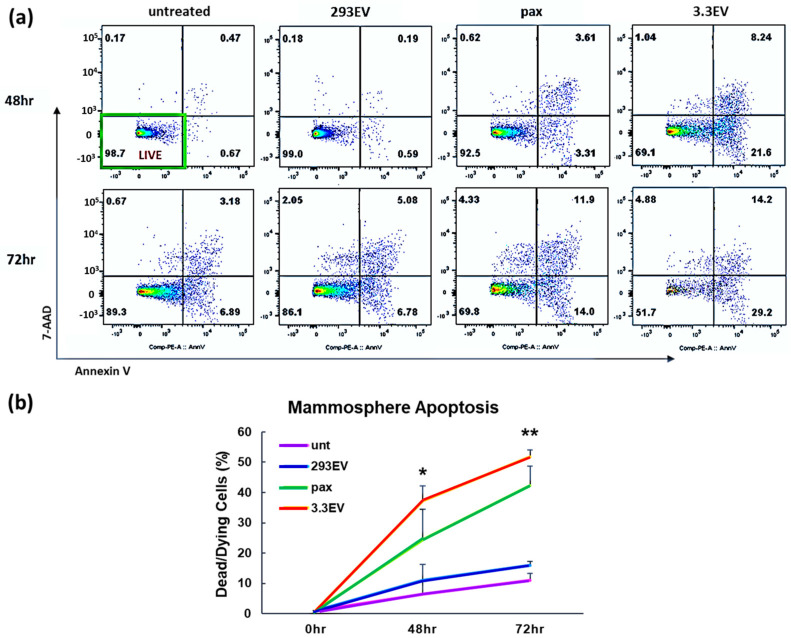
NK3.3EVs induce apoptosis of MCF7 in mammospheres. Mammospheres treated with PBS (untreated or unt), 100 μg/mL HEK293EVs (293EV), 100 μg/mL NK3.3EVs (3.3EV), or 100 nM paclitaxel (pax) for 48 and 72 h were analyzed for annexin V and 7-AAD expression by flow cytometry. An EV concentration of 100 μg/mL corresponds to ~12 × 10^13^ particles/mL. (**a**) Representative scatter plots from a single experiment are shown. Axes for all dot plots are the same. (**b**) The graph represents cumulative apoptosis data from 3 experiments. Dying/dead cell frequency reflects the sum of all annexin V+ and 7-AAD+ quadrants. Mean frequency ± SE. *p*-values determined by comparison of NK3.3EV and pax treatment to HEK293EV treatment. * *p* ≤ 0.05, ** *p* ≤ 0.005.

We investigated whether NK3.3EVs killed MCF7 in mammospheres by induction of apoptosis. Based on previous studies [14,15], we treated mammospheres with PBS, pax, HEK293EVs, or NK3.3EVs for 48 or 72 h. Spheres were dissociated and counted, and all cells were stained with annexin V and 7-AAD to identify cells undergoing early apoptosis (annexin V+) and death (7-AAD+). A representative experiment is shown in Figure 2a. There were more annexin V+/7-AAD ± cells in NK3.3EV-treated mammospheres than in the other cultures at both 48 and 72 h. By 72 h, most of the NK EV-treated mammosphere cells were eliminated, as reflected by the few cells remaining for staining (last dot-plot of Figure 2a). Quantitation from multiple experiments showed that NK3.3EV treatment induced significant apoptotic death compared to HEK293EV treatment (Figure 2b). Pax also induced apoptosis, but to a lesser degree than NK3.3EVs and did not reach the level of significance. A full set of scatter plots for all treatments and time points from a single experiment is shown in Figure S2. These results indicated that NK3.3EVs induced significant apoptosis and cell death of breast cancer cells grown in an in vitro 3D solid tumor system.

### 3.4. NK3.3-Derived EVs Are Taken up and Internalized by Cells Grown in Monolayers, Mammospheres, and 3D Cultures 

**Figure 3:**
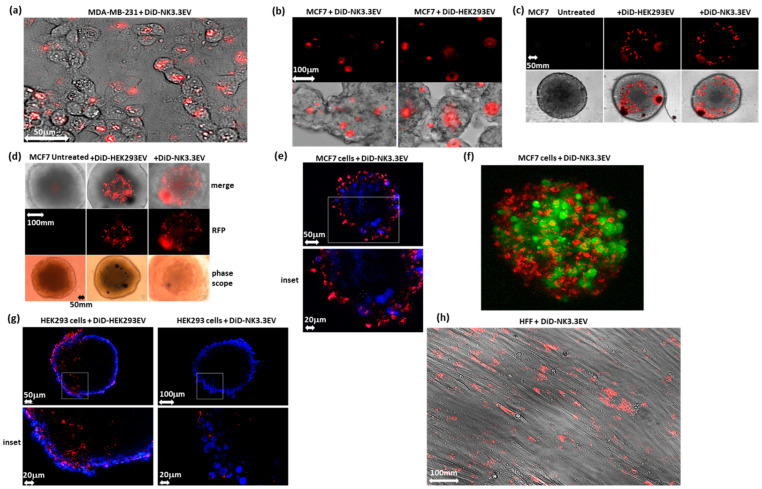
Uptake of NK3.3EVs in monolayer, mammosphere, and 3D cell cultures. (**a**) MDA-MB-231 monolayer cells treated with 50 μg/mL DiD-labeled (red) NK3.3EVs (DiD-NK3.3EV) for 36 h; 200× magnification. (**b**) MCF7 mammospheres treated with 100 μg/mL DiD-NK3.3EVs or HEK293EVs (DiD HEK293EV) for 36 h; 200× magnification. (**c**) Untreated MCF7 3D sphere (DiD only), spheres treated with 10 μg/mL DiD-NK3.3EVs or HEK293EVs for 24 h; 100× magnification. (**a**–**c**) imaged using an inverted fluorescence microscope. (**d**) MCF7 3D spheres imaged using a ZOE fluorescence cell imager after 7 days of treatment. Lower row of images taken using an inverted phase microscope with Olympus color camera; 100× magnification. Black spots are aggregates of DiD stain. (**e**) Image through the center of a GFP-expressing MCF7 3D sphere treated with 10 μg/mL DiD-NK3.3EVs for 24 h; GFP converted to blue in this image; 100× magnification. (**f**) 3D rendering of a GFP-expressing MCF7 sphere treated with 10 μg/mL DiD-NK3.3EV. (**g**) Image through the center of a HEK293 3D sphere treated with 10 μg/mL DiD-NK3.3EVs or HEK293EVs for 24 h; blue: DAPI (nuclei). (**h**) HFF monolayer cells treated with 50 μg/mL DiD-labeled NK3.3EVs for 36 h; 200× magnification.

To evaluate EV uptake by tumor cells, HEK293EVs and NK3.3EVs were labeled with a red fluorescent DiD lipophilic stain and applied to monolayer, mammosphere, and 3D cultured cells. Untreated control cells received DiD alone. After incubation, cells were gently washed to remove EVs that were not tightly cell-bound or internalized. Monolayer cultures of the TNBC cell line MDA-MB-231 treated with DiD-NK3.3EVs showed red punctate staining inside the borders of intracellular compartments (Figure 3a). After 36 h of treatment, cells became vacuolated and showed signs of apoptosis. MCF7 mammospheres were imaged after 36 h of treatment with labeled HEK293EVs or NK3.3EVs. Red punctate fluorescence was observed within mammosphere borders, indicating that the cells internalized both HEK293EVs and NK3.3EVs (Figure 3b).

MCF7 cells cultured in small U-bottom ULA wells formed more tightly structured 3D spheres than the mammosphere clusters in larger flat-bottom ULA wells. Single tight MCF7 spheres were treated with DiD-labeled HEK293EVs or NK3.3EVs. Images taken at 24 h showed punctate fluorescence within sphere footprints (Figure 3c). HEK293EVs appeared to localize closer to the sphere core, while NK3.3EVs appeared more spread out around the sphere periphery. Treated MCF7 spheres were imaged again on day 7 (Figure 3d). Fluorescent signals in MCF7 spheres were still detectable 7 days post-treatment, from either intact EVs or from incorporation of the DiD lipophilic dye into cell membranes. Although MCF7 spheres internalized both HEK293EVs and NK3.3EVs, only NK3.3EV treatment resulted in sphere breakdown, characterized by significant cell debris, vacuolated, empty areas within spheres, and shrinkage of the sphere with loss of sphere definition.

To evaluate the penetration of EVs in 3D cultures, GFP+ MCF7 spheres treated for 24 h with DiD-labeled NK3.3EVs were washed and fixed, and sections through each sphere were imaged by confocal fluorescence microscopy. An image from the center of a representative sphere is shown in Figure 3e. GFP was converted to blue in this image. After 24 h of treatment, DiD-NK3.3EVs penetrated MCF7 spheres, migrating in from the sphere periphery. NK3.3EV penetration was more evident in the 3D rendering of a GFP+ MCF7 sphere (Figure 3f). These images indicated that NK3.3EVs penetrated tumor spheres, resulting in cell death.

EV uptake experiments were also performed using non-tumorigenic cells in monolayer and 3D. HEK293 spheres were incubated with equal concentrations of DiD-labeled HEK293EVs or NK3.3EVs for 24 h, washed, fixed, and analyzed by confocal microscopy. Images through sphere cores indicated that both HEK293EVs and NK3.3EVs penetrated HEK293 spheres (Figure 3g). Examination of multiple images suggested that HEK293 spheres internalized fewer NK3.3EVs than MCF7 spheres but readily internalized their own EVs. We also detected NK3.3EVs in monolayer cultures of human fibroblasts (HFFs), but they had no impact on cell morphology or viability (Figure 3h).

### 3.5. NK3.3-Derived EVs Kill Imatinib-Resistant K562 Cells

**Figure 4:**
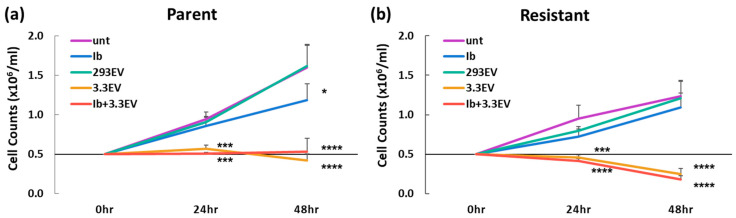
NK3.3EVs kill imatinib-resistant K562 cells. (**a**) Parent and (**b**) imatinib-resistant K562 cells treated with either PBS (unt), 100 µg/mL HEK293EVs (293EV), 100 µg/mL NK3.3EVs (3.3EV), 500 nM imatinib (Ib), or imatinib and NK3.3EVs (Ib + 3.3EV) over 48 h. Cell counts were performed by trypan blue dye exclusion with an automated cell counter at 24 and 48 h. Mean cell counts ± SE; n = 3. *p*-values determined by comparison of NK3.3EV or imatinib-treated cells to HEK293EV-treated cells. * *p* ≤ 0.05, *** *p* ≤ 0.0005, **** *p* ≤ 0.00005.

We investigated whether NK3.3EVs might be a valuable therapeutic for cancer that has become resistant to chemotherapy. Imatinib, a tyrosine kinase inhibitor, is used as a first-line chemotherapeutic treatment for CML patients, but leukemia cells eventually become resistant [23]. To model this in vitro, we developed an imatinib-resistant K562 line. K562 cells were exposed to increasing concentrations of imatinib up to 500 nM over the course of several weeks until resistance was achieved. Consistent with other published findings, resistant K562 cells grew slower than parental cells [28]. Parent and resistant K562 cells were then treated with NK3.3EVs, HEK293EVs, 500 nM imatinib, or the combination of imatinib and NK3.3EVs. NK3.3EV treatment prevented the growth of parent K562 cells and had a cytotoxic effect at 48 h (Figure 4a). Although their growth was slower, NK3.3EVs were even more cytotoxic to imatinib-resistant cells than parent cells (Figure 4b). There was no additive or synergistic effect of imatinib combined with NK3.3EV treatment. HEK293EVs had no effect. Imatinib alone significantly reduced cell numbers by 48 h in parent K562, but much less effectively than NK3.3EV treatment. As expected, imatinib had no effect on resistant K562. Therefore, NK3.3EVs were highly cytotoxic to imatinib-resistant cells and even more effective in killing drug-resistant cells than parent cells.

### 3.6. NK3.3-Derived EVs Induce Apoptotic Death in Imatinib-Resistant K562 Cells

**Figure 5:**
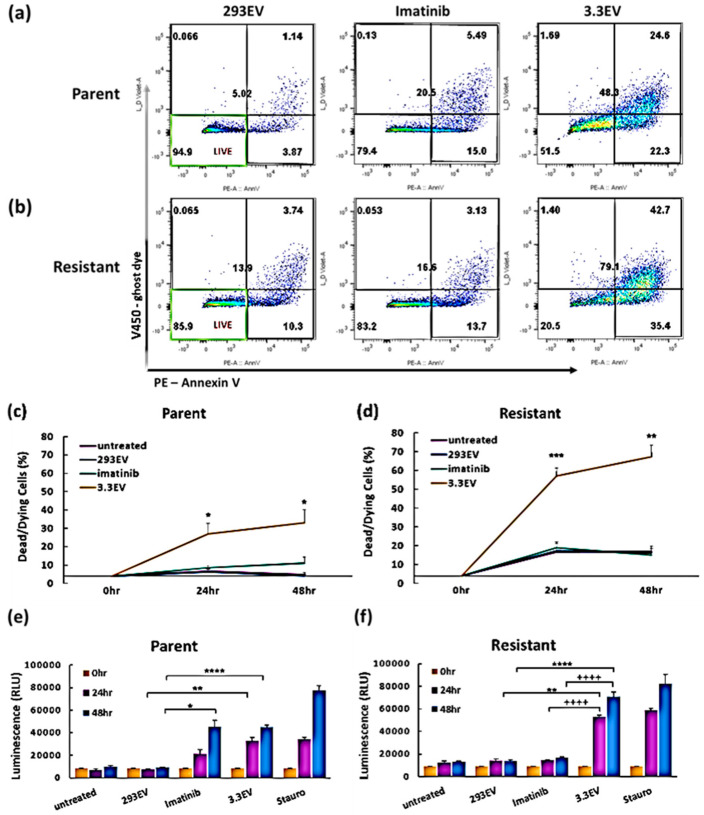
NK3.3EVs induce apoptosis in imatinib-resistant K562 cells. Parent and imatinib-resistant K562 cells treated with PBS (untreated), 100 μg/mL HEK293EVs (293EV), 100 μg/mL NK3.3EVs (3.3EV), or 500 nM imatinib were stained for annexin V and DNA (ghost dye) and evaluated by flow cytometry. Representative scatter plots at 48 h for (**a**) parent and (**b**) resistant cells. Axes for all dot plots are the same as in Figure 2. Graphs of the cumulative apoptosis data at 24 and 48 h are shown for (**c**) parent and (**d**) resistant cells. Dying/dead cell frequency is the sum of all annexin V+ and/or DNA+ quadrants. Mean frequency ± SE; n = 3. *p*-values determined by comparison of NK3.3EV and imatinib-treated cells to HEK293EV-treated cells * *p* ≤ 0.05, ** *p* ≤ 0.005, *** *p* ≤ 0.0005. Apoptosis was measured in (**e**) parent and (**f**) resistant cells at 24 and 48 h after treatment using a caspase-3/7 luminescence assay. Staurosporine (Stauro) (2.5 μM) served as a positive control. RLU: relative luminescence unit. Mean luminescence ± SE; n = 3. *p*-values determined by comparison of NK3.3EV to HEK293EV-treated cells (*) or NK3.3EV to imatinib-treated cells (+). * *p* ≤ 0.005, ** *p* ≤ 0.0005, ****, ++++ *p* ≤ 0.00005.

We next examined whether NK3.3EVs induced death by apoptosis in imatinib-resistant K562. Cells were evaluated for apoptosis by flow cytometry using PE-labeled annexin V to identify early apoptosis and V450-ghost dye to identify dying/dead cells. Representative flow plots of parent (Figure 5a) and resistant (Figure 5b) K562 cells treated for 48 h with either HEK293EVs, imatinib, or NK3.3EVs showed that NK3.3EV treatment increased the frequency of apoptotic cells in both parent and resistant cultures. Cumulative results of multiple experiments demonstrated that NK3.3EV treatment induced significant apoptosis of parent (Figure 5c) and resistant (Figure 5d) K562 cells at both 24 and 48 h. NK3.3EV treatment caused significantly more apoptosis of resistant K562 than parent cells. Imatinib induced a modest level of apoptosis in this assay, which did not reach significance. The full set of scatter plots for all treatments and time points from a single experiment is shown in Figure S3. These results indicated that NK3.3EVs strongly induced apoptosis of imatinib-resistant K562 cells.

Previous work by our laboratory determined that NK3.3EVs induced significant caspase-3 and -7 activity in K562 cells as early as 1 h post-treatment and continued to increase over 72 h [14]. Here, we determined whether NK3.3EVs induced caspase-3/7 activity in imatinib-resistant K562. NK3.3EV treatment produced highly significant caspase-3/7 activity in both parent (Figure 5e) and resistant (Figure 5f) K562. NK3.3EVs activated twice as much caspase-3/7 in resistant than in parent K562 cultures. This paralleled the annexin V/ghost dye staining results above. In the caspase-3/7 assay, we detected apoptosis in imatinib-treated parent K562 cells at 48 h. As expected, imatinib had no effect on resistant K562. These results confirmed that NK3.3EVs strongly induced apoptosis of resistant K562 that no longer responded to imatinib and, in fact, were even more effective in killing drug-resistant than non-resistant K562 tumor cells.

### 3.7. NK3.3-Derived EVs Reduce the Number of Imatinib-Resistant K562 Cells with a CSC-like Phenotype 

**Figure 6:**
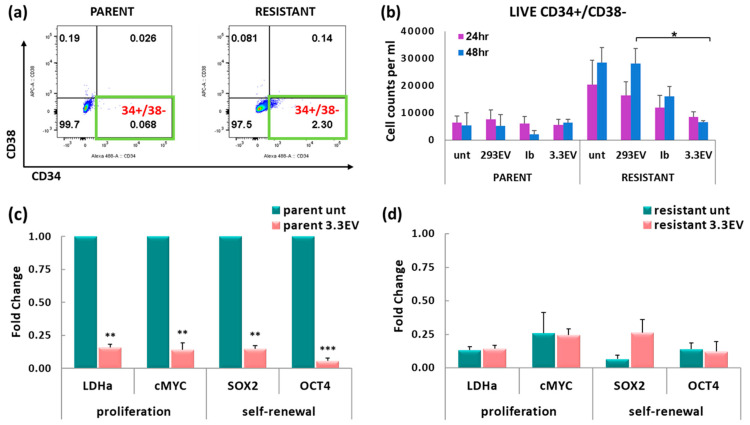
NK3.3EV treatment reduces the number of CSC-like K562 cells. (**a**) Representative scatter plots show the gating strategy for identifying cells with the CD34+/CD38− CSC-like phenotype (lower right quadrant). (**b**) Cells were treated with PBS (unt), 100 μg/mL HEK293EVs (293EV), 100 μg/mL NK3.3EVs (3.3EV), or 500 nM imatinib (Ib) for 24 and 48 h. CSC frequencies and manual counts were used to determine the absolute numbers of CD34+/CD38− cells after each treatment. Mean cell counts ± SE; n = 3. *p*-values determined by comparison of NK3.3EV to HEK293EV-treated cells. * *p* ≤ 0.05. (**c**) Parent and (**d**) imatinib-resistant K562 cells treated with PBS (unt) or 100 μg/mL NK3.3EVs (3.3EV) for 24 h were evaluated for expression of proliferation and tumor-promoting genes by qPCR. Target gene expression was normalized to expression of housekeeping gene 18S rDNA. Mean fold change ± SE and *p*-values were determined by comparison to untreated parent K562. n = 3. ** *p* ≤ 0.005, *** *p* ≤ 0.0005.

The CSC-like subpopulation of CML is recognized as having a CD34+/CD38- phenotype [29]. Parent and imatinib-resistant K562 cell cultures were stained to identify CD34 and CD38 surface marker expression by flow cytometry. Representative flow scatter plots highlight the CSC-like subpopulation in K562 cells in the lower right quadrant (Figure 6a). Overall, resistant K562 cultures had a larger population of CSC-like cells than parent K562 cultures.

Parent and resistant cells were untreated or treated with HEK293EVs, NK3.3EVs, or imatinib for 24 and 48 h. Live cell frequencies and manual total cell counts were used in conjunction with corresponding CD34+/CD38− frequencies to determine the absolute number of CD34+/CD38− cells in each condition from multiple experiments. NK3.3EV treatment of resistant K562 significantly reduced the CSC-like population compared to HEK293EV-treated cells at 24 and 48 h (Figure 6b). We were not able to determine with confidence the effect of NK3.3EV treatment on CSC-like numbers in the parent cultures due to their very low numbers of CSC-like cells. Scatter plots from a full experiment are shown in Figure S4. These results indicated that NK3.3EV treatment significantly reduced the CSC-like subpopulation within the drug-resistant K562 tumor cell culture.

### 3.8. Treatment of K562 with NK3.3-Derived EVs Reduces Tumor-Promoting Gene Expression 

In addition to using the CD34+/CD38- phenotype as a marker of CSC, gene expression by qPCR was performed to determine the impact of NK3.3EV treatment on the levels of proliferation-associated genes lactate dehydrogenase (LDHa) and cMYC, and self-renewal-associated genes SOX2 and OCT4. After 24 h of NK3.3EV treatment, the expression of all four genes was significantly lower (by 5–10-fold) in parent K562 cultures (Figure 6c). Expression of these genes was intrinsically much lower in resistant cultures than in parent cultures; we observed no significant difference in gene expression between untreated and NK3.3EV-treated imatinib-resistant cells (Figure 6d). These results indicated that NK3.3EV treatment eliminated CSC-like cells and/or suppressed tumor-promoting gene expression in K562 parent cultures.

## 4. Discussion

A major challenge in the effort to successfully treat cancer is the emergence of tumor cell subpopulations that become resistant to therapy. Phenotypic plasticity, genetic alterations, immunoediting, and other mechanisms allow tumor cells, especially the CSC population, to enhance their survival mechanisms and develop resistance [30]. Another challenge to fighting cancer is the difficulty in developing treatments that penetrate and reach cells deep within tumor tissue. The tumor microenvironment, which is acidic, hypoxic, and immunosuppressive, is a barrier to effective cellular and immune therapies. However, this environment may be optimal for EVs to function. Unlike cells, EVs have a high extravasation capacity, greater ability to access tumor cells, and remain fully active [22]. It has been suggested that EVs more readily fuse to tumor cells under acidic conditions [31]. Our study was designed to test the ability of NK3.3EVs to circumvent some of the obstacles to treatment by evaluating their penetration into 3D solid tumor cultures and their capacity to kill drug-resistant tumor cells and eliminate cells with a CSC-like phenotype.

We demonstrated that NK3.3EVs penetrate breast cancer cells grown in 3D cultures. They kill tumor cells in 3D cultures by apoptosis as effectively or better than tumor cells grown in monolayers. Mammosphere and 3D cultures partially reproduce elements seen in solid tumors in situ, including cell communication, response to stimuli, proliferation, gene expression, phenotypic plasticity, nutrient gradient modulation, and gas exchange to cells in the core [4,5,6]. The power of NK3.3EVs to kill solid tumor cells in a 3D format indicates that NK3.3EVs can circumvent some of the physiological barriers they would encounter in vivo. We previously demonstrated that the intratumoral injection of NK3.3EVs induced significant apoptosis of MDA-MB-231 breast cancer cells in vivo [14]. Collectively, this makes NK3.3EVs a promising new cancer treatment option.

The question of specificity of NK3.3EV uptake remains open. We can detect uptake of NK3.3EVs in non-tumorigenic, healthy cells, but this does not affect their growth or viability. The amount of EV internalization may be a factor; our studies to date suggest that MCF7 breast cancer spheres internalize more NK3.3EVs than HEK293 spheres. Analyses are in progress to more fully quantitate NK3.3EV uptake and penetration in tumor and non-tumorigenic cells. It will be important to determine whether NK3.3EV-mediated killing of tumor cells but not healthy cells is due to differences in EV binding/signaling, the level of EV uptake, or differences in EV processing and/or cargo release within cells after internalization. Most published studies report NK EV uptake by tumor cells without evaluating uptake by non-tumorigenic cells [8,16,17,18]. The remaining studies are mixed regarding the specificity of EV uptake. Samara et al. [19] showed that NK EVs were selectively taken up by leukemia cells but not by healthy B cells. Other studies found that NK EVs were internalized by normal cells, but they did not induce cytotoxicity, similar to what we observe [8,14,15,20]. Two studies found that blocking DNAM1 on the surface of NK EVs partially inhibited their tumoricidal activity [16,21]. Another reported partial inhibition of NK EV-mediated apoptosis of tumor cells by blocking NKG2D [17]. It is likely that there are many receptor–ligand interactions between NK EVs and their targets; some may be tumor as well as tumor cell-specific. NK cells employ multiple receptors and cytotoxic molecules to kill target cells. EVs derived from NK cells have these same components, suggesting that NK EVs may interact with and kill tumor cells by multiple mechanisms [12,20]. NK3.3 and other NK EVs also contain microRNAs with tumor suppressor activity [11,14,20]. These may contribute to the specificity of NK EVs for tumor killing.

As a model of drug resistance, we generated an imatinib-resistant K562 cell line. K562 has the characteristic Philadelphia chromosome, which defines CML [32]. The Philadelphia chromosome is created by translocation of the ABL gene of chromosome 9 with the BCR gene of chromosome 22 (t(9;22)), resulting in the BCR-ABL fusion gene and the Bcr-Abl fusion protein. Constitutive Bcr-Abl tyrosine kinase activity prevents apoptosis by inhibiting cytochrome c release from mitochondria, upregulating pro-survival proteins c-FLIP and Bcl-xL, and phosphorylating pro-apoptosis protein, Bim, marking it for degradation. Imatinib inactivates Bcr-Abl activity by binding to the catalytic site of the protein, resulting in the restoration of apoptosis [33,34,35,36]. The generation of BCR-ABL point mutations that prevent imatinib binding contributes to the development of imatinib resistance [23]. 

Imatinib resistance was established in K562 at a concentration of 500 nM. This dose inhibited the expansion of parent cells in vitro, induced apoptosis, and was within the IC_50_ range reported in other in vitro studies [23]. NK3.3EVs were more cytotoxic to drug-resistant cells than parent K562 cells. NK3.3EVs also induced greater caspase-3/7 activity and apoptosis in drug-resistant cells than in parent cells. This indicates that NK3.3EV treatment is highly effective in killing cells that are resistant to front-line imatinib chemotherapy. Drug-resistant cells retained their ability to die through the induction of intrinsic apoptosis mediated by NK3.3EV treatment. Imatinib at 500 nM slowed proliferation and induced caspase-3/7 activity in parent cells but was not as effective as NK3.3EV treatment. The combination of imatinib and NK3.3EVs had no additive effect. Studies are ongoing to examine the anti-tumor activity of NK3.3EVs in a variety of drug-resistant tumors.

This and our previous study demonstrated that NK3.3EVs kill tumor cells by apoptosis, as measured by increased frequencies of annexin V+ cells, increased caspase-3/7 activity, and cleavage of caspases-3, 7, and 9 [14,15]. NK EVs with higher levels of perforin and granzyme B have been reported to have more potent anti-tumor activity than EVs with lower amounts, suggesting that these proteins are primary mediators of cell death [9,37]. Pan-caspase inhibitors partially but significantly reduce NK EV-mediated tumor killing [8,9,10]. Of the multiple proteins (including the cytokines IFN-γ and TNF-α) and microRNAs packaged within NK EVs, it is likely that several, working alone or together, participate in the killing process. Studies are in progress to further identify the role(s) of various EV constituents in NK3.3EV-mediated tumor cytotoxicity.

We used the CD34+/CD38- surface marker phenotype to identify leukemic stem cells [29,38]. Long-term imatinib exposure increased the CD34+/CD38- subpopulation in resistant K562 compared to parent cultures, recapitulating results seen in other studies where K562 cultures shifted to a more stem-like phenotype after they developed drug resistance [28,39,40]. NK3.3EV treatment significantly reduced the imatinib-resistant CSC-like subpopulation. 

Yong et al. [41] showed that replicating CD34+ patient-derived CML cells were more susceptible to NK killing than CD34− cells or quiescent CD34+ cells. NK receptors responsible for selective recognition of CSC-like cells in solid tumor models were identified as NKG2D, NKp44, and NKp30. Lower expression of MHC-I on CSC and de-differentiation were also cited as reasons for greater selectivity [42,43]. These NK receptors are present on NK3.3 cells and have been found on NK-derived EVs [10,13]. K562 cells express the NKG2D ligands MIC-A and -B, and ULBP family proteins [44,45]. It is unclear whether a grouping of these interactions results in more NK3.3EV uptake in CSC-like cells, or whether these receptors and ligands affect the function of NK3.3EVs after entering tumor cells. Investigations of the role of NK receptors in NK3.3EV-mediated killing of CSC are underway.

In addition to using the CD34+/CD38 phenotype to identify CSC-like cells, we also examined the effect of NK3.3EVs on the expression of genes associated with CSC-like cells and tumor progression. The oncogene, cMYC, which is often overexpressed in cancer, is a transcription factor that targets numerous genes associated with metabolic activity, proliferation, differentiation, and stem cell renewal. LDHa expression, upregulated by cMYC, enhances aerobic glycolysis and promotes tumor expansion [46,47]. The transcription factors SOX2 and OCT4 participate in embryogenesis and stem cell maintenance and contribute to CSC formation, tumor growth, and metastasis upon inappropriate cytoplasmic or nuclear localization [47]. Reduced LDHa and cMYC expression in this study complements our previous findings that low doses of NK3.3EVs inhibit tumor cell proliferation [14,15]. We also saw a significant reduction in SOX2 and OCT4 gene expression in NK3.3EV-treated cultures. This likely reflects NK3.3EV-mediated killing of CSC-like cells, but it is also possible that NK3.3EV treatment affects CSC-associated gene expression.

Methods for optimizing NK3.3EV production and isolation are the necessary next steps towards their clinical utilization. We previously compared the anti-tumor activity of NK3.3EVs prepared by PEG precipitation with ultracentrifugation and found they generated equivalent particle sizes and bioactivity [14]. However, ultracentrifugation is labor-intensive, and EV yields are low. Tangential flow filtration also generates equivalent particles as PEG precipitation (Figure S1) and similar anti-tumor activity. Combining PEG precipitation with other separation methods, such as size exclusion chromatography, also produces EV particles with anti-tumor activity, but the yields drop precipitously.

Collectively, we have shown that NK3.3EVs are powerful inducers of cell death in leukemia cells and in solid tumor cell lines in both monolayer and 3D cultures. They are especially cytotoxic to imatinib-resistant cells, circumventing their resistance mechanism(s). NK3.3EVs also significantly reduce the CSC-like CML subpopulation, which evades treatment, leading to tumor dissemination and relapse. Importantly, unlike traditional chemotherapy, NK3.3EVs are not toxic to normal, healthy cells. The current therapeutic modalities for multiple cancer types, especially those that lack effective, curative treatments. 

## 5. Conclusions

We demonstrate here that EVs derived from NK3.3 cells have the potential to be a new form of cancer treatment. We show that NK3.3EVs penetrate and kill solid breast tumors in an in vitro 3D tumor model. We also determined that NK3.3EVs can circumvent resistance mechanisms that allow tumor cells to escape standard therapeutic modalities. NK3.3EVs induce the death of imatinib-resistant K562 chronic myeloid leukemia cells and eliminate the CSC-like tumor subpopulation responsible for disease recurrence. A major advantage of using NK3.3EVs to treat cancer patients is that, unlike standard chemotherapy, NK3.3EVs are not toxic to normal, healthy cells. NK3.3EVs can be produced in large quantities, frozen, and thawed without loss of function. This should make EV therapy less expensive than cellular therapies and more accessible to a greater number of patients. 

Preliminary preclinical animal studies have shown that NK3.3EVs kill leukemia and breast cancer cells in vivo [14]. Further experiments are in progress to extend our studies to additional types of cancer. Early results suggest that NK3.3EVs have potent killing activity against many different tumor cell lines and primary patient tumor cells, including those that are relatively resistant to NK cell-mediated cytotoxicity. Although the clinical use of NK3.3EVs, especially for patients with chemo-resistant tumors, is in the first phase of development, our initial results are highly encouraging and the subject of continued investigation.

## Data Availability

Original data will be made available upon request.

## Supplementary Materials

The following supporting information can be downloaded at https://www.mdpi.com/article/10.3390/cancers16010090/s1, Figure S1: NTA analysis of HEK293EVs isolated by PEG precipitation and NK3.3EVs isolated by either PEG precipitation or by tangential flow filtration (TFF). Figure S2: Full dataset of a representative experiment demonstrating NK3.3EV-induced apoptosis of MCF7 mammospheres; Figure S3: Full dataset of a representative experiment demonstrating NK3.3EV-induced apoptosis of parent and drug-resistant K562 cells; Figure S4: Representative experiment demonstrating the effect of NK3.3EV treatment on the frequency of CD34+/CD38− CSC-like K562 cells.
