# Supplementary material for: NK3.3-Derived Extracellular Vesicles Penetrate and Selectively Kill Treatment-Resistant Tumor Cells
Source: Cancers (Basel). 2023 Dec 23;16(1):90. doi: 10.3390/cancers16010090 (PMC10778188; doi:10.3390/cancers16010090)
Supplement: Supplementary file 1 [file cancers-16-00090-s001.zip › cancers-2738307-supplementary.pdf]

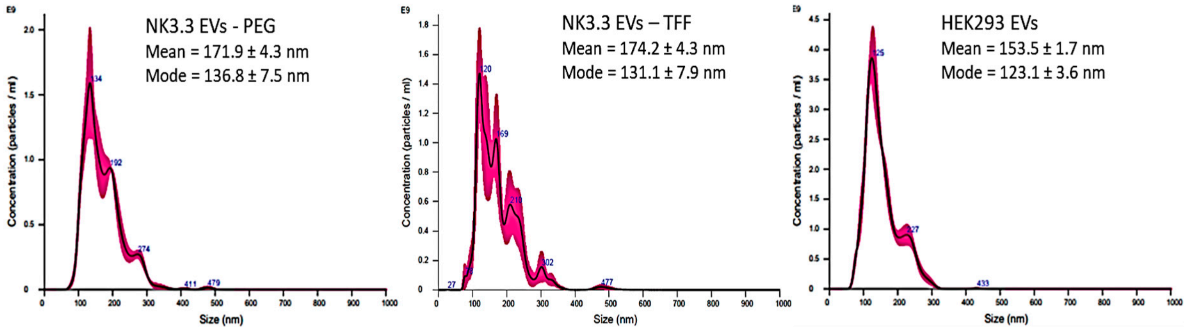

Figure S1. NTA analysis of NK3.3EVs isolated by PEG precipitation (PEG), tangential flow filtration (TFF) and HEK293EVs isolated by PEG precipitation.

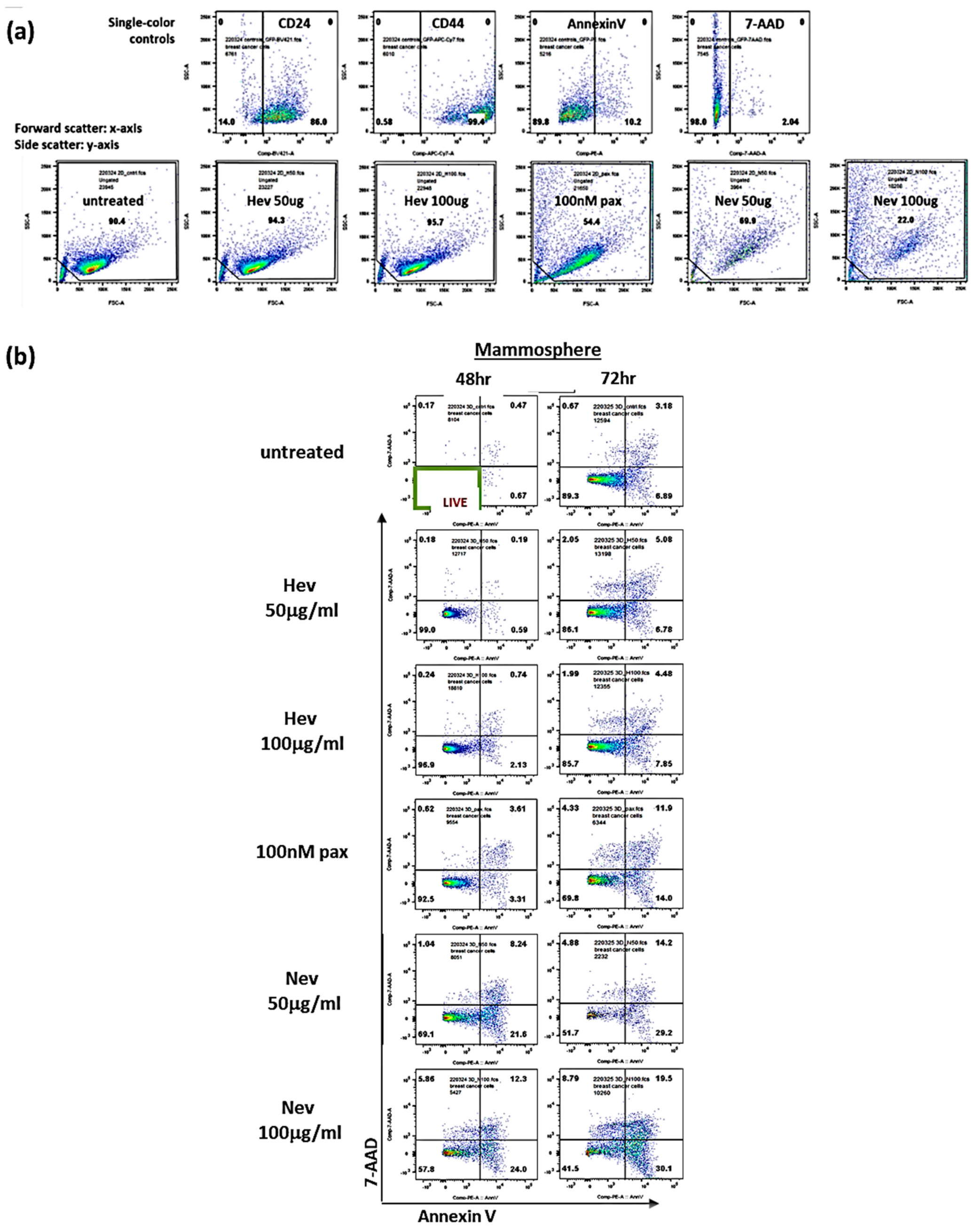

Figure S2. Full dataset of a representative experiment demonstrating NK3.3EV-induced apoptosis of MCF7 mammospheres. (a) Top row: plots show gating

strategy for single-color staining of annexin V and 7-AAD by flow cytometry. Bottom row: plots show gating strategy for removing debris using forward scatter (FSC-A) on the x-axis and side scatter (SSC-A) on the y-axis. (b) MCF7 mammospheres treated with PBS (untreated), 50 or 100µg/ml HEK293EVs (Hev) or NK3.3EVs (Nev) for 48 and 72 hours. Paclitaxel (pax) (100nM) served as a positive control. Plots from a single experiment show the frequency of cells undergoing apoptosis and late apoptosis/necrosis.

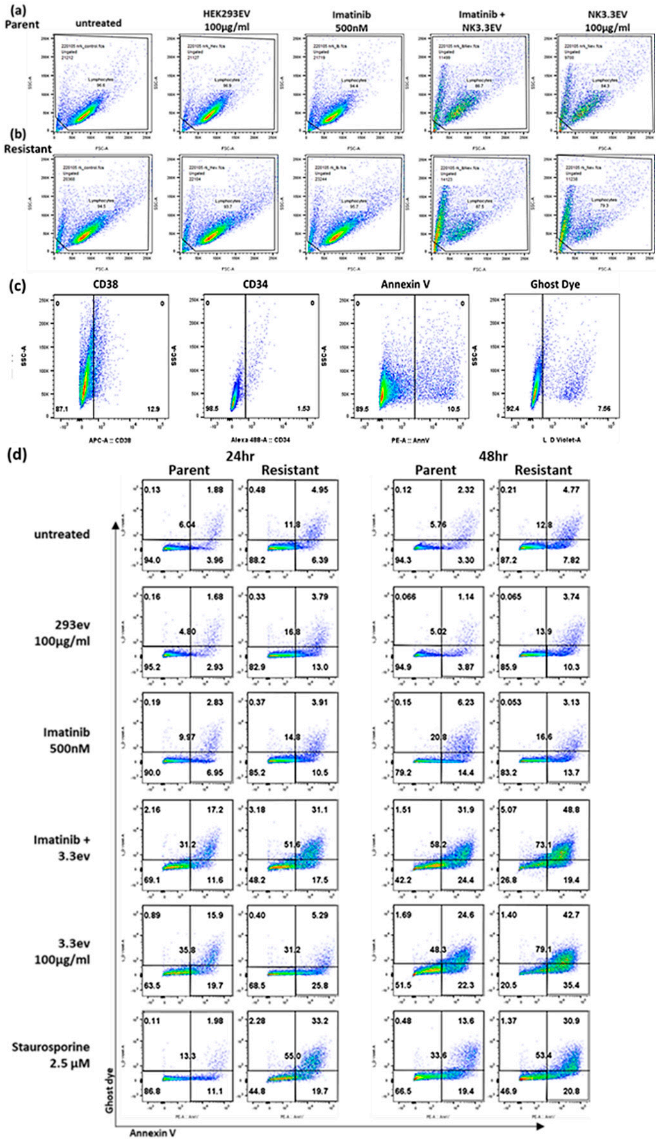

Figure S3. Full dataset of a representative experiment demonstrating NK3.3EV induced apoptosis of parent and drug resistant K562 cells. Parent and drug resistant K562 cells treated with PBS (untreated), 100µg/ml HEK293EVs (293ev) or NK3.3EVs (3.3ev), 500nM imatinib, 500nM imatinib+ 100µg/ml NK3.3EV, or 2.5µM staurosporine for 24 and 48 hours. Cells were stained with annexin V and ghost dye and analyzed by flow cytometry. Plots show gating strategy for removing debris using forward scatter (FSC-A) on the x-axis and side scatter (SSC-A) on the y-axis for (**a**) parent- and (**b**) resistant cells at 24 hours, (**c**) gate set-points for CD38, CD34, annexin V and ghost dye, and (**d**) apoptosis scatter plots for all treatment conditions for parent and resistant cells at 24 and 48 hours. .

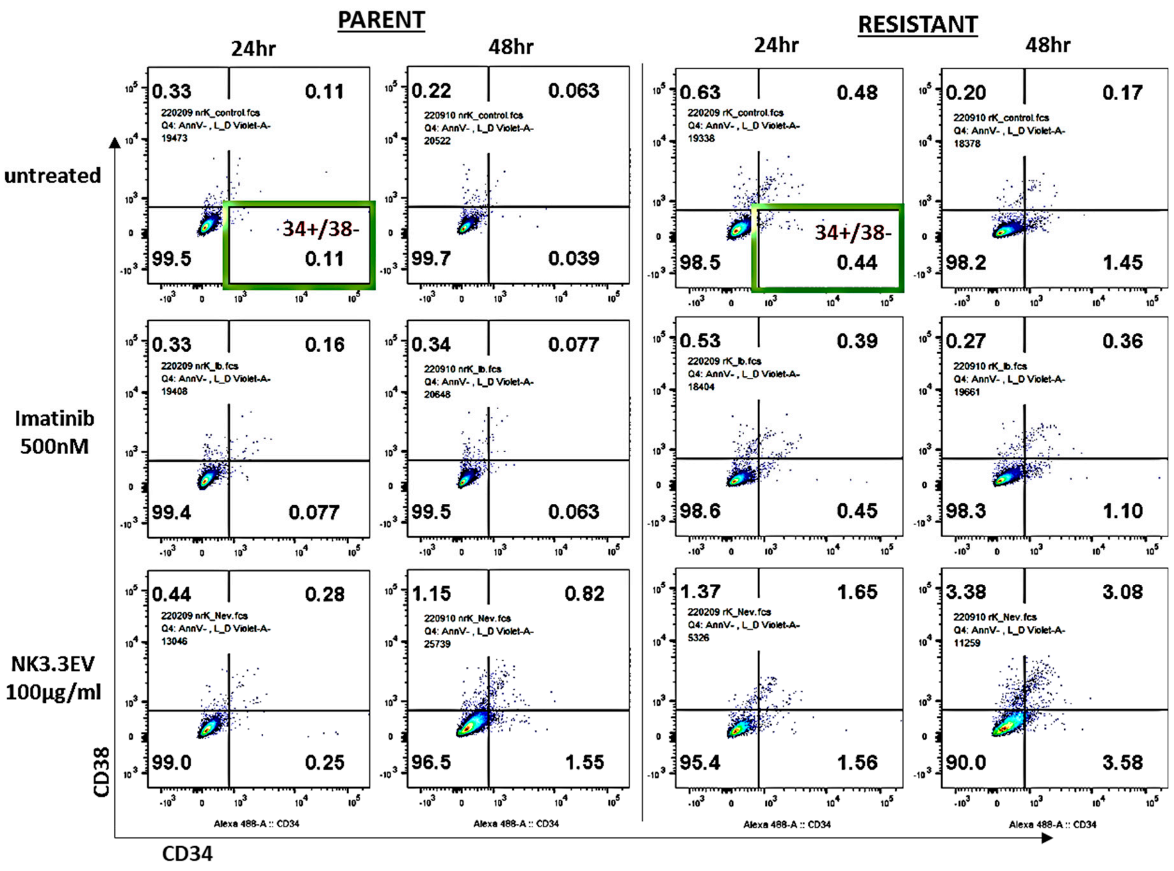

Figure S4. Representative experiment demonstrating the effect of NK3.3EV treatment on the frequency of CD34+/CD38- CSC-like K562 cells. Cells treated with PBS (untreated), 500nM imatinib or 100µg/ml NK3.3EVs for 24 and 48 hours. Scatter plots show the frequency of CD34+/CD38- cells in parent and drug resistant cells. Cells with the CSC-like phenotype identified in the lower right quadrant with their frequency.
